# Pathology and Practice of Medicine

**Published:** 1860-09

**Authors:** Richard J. Dunglison

**Affiliations:** Physician to the Burd Orphan Asylum and to the Albion Society


### PATHOLOGY AND PRACTICE OF MEDICINE.

BY RICHARD J. DUNGLISON, M.D.,
PHYSICIAN TO THE BURD ORPHAN ASYLUM AND TO THE ALBION SOCIETY.

### I.—*A fatal Case of Trichina Spiralis.* (London Medical Times and Gazette.)

A small species of entozoa occasionally exists in the muscles of voluntary motion, to which the name *trichina spiralis* has been given. The minute cysts which constitute the trichinæ are usually of an oblong shape, and in size and color have considerable resemblance to young pediculi. We here condense the history of their existence in a young girl, admitted into the wards of the Dresden Hospital, who had presented symptoms not unlike those of rheumatic affections, accompanied with many of the concomitants of typhoid fever; a very large number of these animalculæ were discovered, many arranged spirally in the structure of the muscle, while others were extended. They were found to exist also in all the striated or voluntary muscles, but the structure of the heart was free from them. When the intestines were examined, the trichinæ were discovered in vast numbers.

The previous history of the case showed that the patient had been living in a butcher's establishment, in which smoked ham and sausages had been prepared but a day or two before she was taken sick, from two pigs slaughtered there; and it was considered probable that the girl had eaten some of the food. On examining portions of the sausages and ham, trichinæ were visible in great numbers. The proprietor of the establishment was affected with symptoms similar to those of his maid-servant, but death did not supervene as in her case. One curious fact was revealed in this case in regard to the reproduction of these animalculæ. The female trichinæ contained in the central portions of their bodies large numbers of well-developed embryonic trichinæ, thus showing that these parasites are viviparous.

### II.—*Paralysis of the Bronchial Muscles.* By JAMES F. DUNCAN, M.D., etc. (Dublin Quarterly Journal of Medical Science, May, 1860, p. 499.)

So much attention has been paid, Dr. Duncan thinks, to spasmodic contraction of the muscular fibres connected with the air-passages, that the opposite condition of impaired contractile power has been too much disregarded. The minuteness of these muscles may have induced medical men to underrate their importance as aids in the proper performance of the function of respiration; and inasmuch as they are not voluntary in their nature, the loss of their contractile energy is not so readily appreciated. The elasticity of the structure of the lung would counteract any injurious effects that would certainly follow, should paralysis of these muscular fibres be attended by collapse of the air-passages. One great difficulty in the recognition of affections of the muscular portions of the respiratory apparatus is that, unlike other parts of the lung, morbid changes of the muscular structure "defy alike the scalpel of the anatomist and the lens of the microscopist."

Yet there exist strong reasons for believing, according to Dr. Duncan's views, that long-continued inflammatory action in the mucous membrane of the bronchi may impair or destroy the contractility of the muscular fibres which lie in close contact with the under surface of the inflamed bronchi. A case is detailed in illustration of this theory, in which he thinks the symptoms may be rationally considered as explicable according to the views above mentioned. Without entering into a minute history of the case, the peculiarity of the dyspnœa, from which she suffered greatly, may well claim attention here. Each act of respiration was to a certain extent voluntary, for the involuntary muscles were not able, without assistance, to maintain the requisite expansion of the chest. Great disproportion existed between the duration of inspiration and of expiration, the latter being fully three times as long as the former—a fact which was visible to the eye of an observer. This prolonged expiratory murmur was probably due, Dr. Duncan thinks, to impaired action of the muscular fibres, and he regards the extension of the effort of expiration as pathognomonic of paralysis of the muscular fibres of the bronchial tubes.

It is an interesting portion of the history of the case, which seems somewhat confirmatory of the view taken by its describer, that all treatment failed to give relief, until the extract of nux vomica, an agent which, he considers, "stimulated the muscular apparatus of the pulmonary system, and, by so doing, removed a mechanical impediment to the proper performance of the respiratory functions," was prescribed. Ten days after the commencement of this mode of treatment, she was sufficiently improved to be discharged from the hospital.

III.—*The Scrofulous Conformation or Diathesis; based principally on the Study of the Skeleton.* By Dr. T. G. HAKE. (Medical Times and Gazette, April 21, 1860.)

The peculiar conformation of body, or diathesis, considered distinct from the scrofulous disease, has only been described by modern writers, while scrofula itself is one of the oldest affections on record. It is the hereditary element on which scrofula, tuberculosis, and rickets are engrafted. The characters of the scrofulous type of conformation, says Dr. Hake, refer to the shape of the cranium, the jaws, the limbs, and to the symmetry and growth of the body in general. It has been found that while several of these and other characters are abnormal in relation to the European, or so-called Caucasian, they are distinctive of the inferior race; that all these characters are discoverable in the fœtus or the children; that, as a rule, the features of the scrofulous conformation, and of the inferior varieties of mankind, exist normally in the embryonic and infantile structures of the European or higher type of man. Twenty-two propositions are stated by the author as the conclusions at which he had arrived, all of which are fully illustrated by tables and reports. From these we select a few interesting facts and theories. Comparison of the cranium and long bones of an adult of the scrofulous type with those of the fœtus and child, displays their common characters. During the period of intra-uterine life, and afterwards, the cranium is large in proportion to the other parts of the skeleton; the frontal portion is low and narrow, etc. These characters gradually give way after birth to those of the Caucasian type, except in such children as inherit the scrofulous diathesis, in whom they persist throughout life. The crania of the fœtal series (Caucasian type) and those of the adult inferior varieties of man correspond, by admeasurement, in their relative diameters. The cranium of the adult Ethiopian and Mongolian finds its parallel constantly in the European fœtus.

So also we find the pelvis of the fœtus having characters which agree with those of rickety subjects in the same structure and with those of certain adult varieties of man. Thus, the pelvis is oblong, in a direction from the symphysis pubis to the sacrum, in the European fœtus, also in the male negro, the New Hollander, the male and female Buschisman, and the male Polynesian (Hunterian Collection.) In the fœtus of the dwarf, the female Buschisman, etc., the ilia are upright; in the adult European they are expanded and hollowed; and so on. Such coincidences occur also, according to Dr. Hake, in the predominance of the organic over the earthy constituents of bone, as characteristic of rickets and of the osseous system in the embryo; in the direction which the neck of the femur assumes to the shaft in each; the gradual transformation of the fœtal cranium from the Ethiopian type, which it assumes during the early weeks of embryonic life, to the Mongolian,—then, by degrees, to that of the Caucasian; the large size of the ends of the ribs, both in the fœtus and in rickety subjects, etc.

The prolonged infancy of our species has to be taken into account in explaining the blue eye of adults. An opportunity having occurred for examining the negro fœtus in relation to these points, the iris was found to be of a pale color, grayish blue; the hair was black; there was an Ethiopian cast of feature; the nose was depressed, the lips were thick, etc. The features themselves were scarcely distinguishable in shape from those of a European fœtus of from four to six months, and in their character and type were such as are daily observable in European adults who have the immature organization which is allied to scrofula or tuberculosis, whether those diseases be present or absent.

Dr. Webster, in reply to Dr. Hake, stated that throughout Spain, where a blue eye and light hair were scarcely ever seen, while the natives appeared unusually dark and Moorish-looking, strumous maladies existed to a great extent. Scrofula was very common also among dwarfs, and doubtless frequently made people dwarfs; but, excepting the Swedes, there was not a better formed or more gigantic race of men than the Spaniards, especially in Castile, and yet scrofula often existed. The author had evidently attributed to race results which arose from food and other causes; since it was found that in nations living chiefly upon vegetable diet, as in Spain, and no doubt also in Italy, where dark complexions also prevailed, scrofula was particularly prevalent, and that as they became an animal-food-eating people, scrofulous diseases proportionately diminished.

Dr. Hake seems to have considered the blue eye merely as a feature of immature development, while it should be borne in mind also that it was not scrofula as a disease which had been brought forward, but merely the conformation on which it was based or engrafted. Otherwise he could have shown how largely scrofula exists in many of the dark races, particularly in the Australian. Incompatibilities and discrepancies seem to exist, which might suggest doubts whether the physical development of animals would bear out the author's conclusions; but these may lead to a further consideration of the subject.

IV.—*Sudden Death, without any adequate Post-mortem Appearances.* By J. ZACHARIAH LAURENCE, F.R.C.S., etc. (British Medical Journal, May 19, 1860.)

In this case, a house-maid, no symptoms were observable before death except earache, unusual indolence and stupidity, and occasional faintness; death supervened suddenly, and post-mortem examination revealed no phenomena of sufficient consequence to account for the fatal result. Mr. Laurence states that all the usual phenomena observed in cases of sudden death were absent. In the *Annales d'Hygiène Publique* for 1838, M. Devergie published an interesting paper, "*De la Mort Subite,*" in which one cause of sudden death is that by syncope, which he had found to occur three times in forty cases. It appears to be identical with the "idiopathic asphyxia" of Chevalier. In the three cases detailed by M. Devergie, the prominent appearances discovered on post-mortem examination were: 1, absence of all congestion of the organs; 2, the normal state of all the organs; 3, the existence of about an equal quantity of blood in the right and left cavities of the heart, regard being had to their respective sizes; 4, perhaps the coagulation of the blood in the fibrinous state.

In the present case, the stomach contained an undigested meal which the patient had partaken of stealthily, and therefore, probably, hastily; and a patch of injection existed in the coats of the organ. These conditions of the stomach may not unreasonably be connected with the syncopal affection, and the case may be regarded as one of a mixed nature, presenting signs of syncopal asphyxia, as well as of apoplexy. Yet the phenomena were totally inadequate to account for the suddenly fatal result.

V.—*Influence of Warm Climates on Phthisis.* By PROF. FORGET, of Strasburg. (Gazette Hebdomadaire de Médecine et de Chirurgie, June 8, 1860.)

The medical periodical from which we translate and condense the following speculations in regard to the influence of climate on pulmonary consumption, has had access to an unpublished work by Prof. Forget, entitled "*Principes de Thérapeutique Générale et Spéciale ; Nouveaux Eléments de l'Art de Guérir.*" The eminent author of this work refutes the idea advanced by a writer whose researches were crowned by the *Académie des Sciences*, that a residence in warm climates favored the production and development of phthisis, founding his view on the theory that debility is influential in the generation of tubercular consumption, and that warmth engenders debility.

The conclusions at which M. Forget arrived may be briefly summed up. It is not by the debility which they cause that warm climates are favorable to phthisis; it is, on the contrary, by the stimulation which the extreme heat exerts on the respiratory passages which are chronically affected. Warm climates are only unfavorable to consumptives from the excess of solar heat. But when this excess is counteracted by appropriate measures, a residence in a warm climate is attended with most happy results, a fact confirmed by the testimony of numbers of invalids who owe to emigration thither the prolongation of their life, and sometimes their cure. It is this which makes the neighborhood of the tropics more favorable than residence under the equator, and Madeira or Southern Africa than Bengal or Brazil.

If, in place of braving the extreme solar heat, the sick keep in the shade, in an apartment protected from the invasion of the exterior atmosphere; if they choose as hours for walking only the first and last hours of sunshine, they then receive all the benefits of the warm season without experiencing its inconvenience. M. Forget refers to the fact that observations have been made and statistics collected among the poor soldiers, and unfortunate marines, who were obliged to work actively under a vertical sun, and inferences been drawn erroneously from these facts as proofs of the pernicious influence of warm climates on those suffering from tuberculosis.

VI.—*On the use of the Ophthalmoscope as a help to Diagnosis in Diseases of the Nervous System.* By John Ogle, M.D., etc. (Med. Times and Gazette, June 9, 1860.)

The valuable instrument, whose original object, when invented, was the diagnosis of certain affections of the eye, has received an extension of its usefulness to the examination of other diseases which are not primarily diseases of the eye itself. Dr. Ogle endeavors to make it subservient to the interests of diagnosis in certain diseases of the nervous system. He thinks that a deranged condition of the cerebral circulation may be mirrored forth by the ascertained condition of the vessels and inner coats of the eye in those cases in which this disorder is dependent upon disturbance of vaso-motor influence by reason of interference with distant parts of the sympathetic nervous system. Clinical cases, as well as the results of accidental injuries and experimental operations upon the sympathetic system, seem to show that giddiness, dimness of vision, deafness, and other such symptoms coincident often with intra-thoracic aneurism and other tumors about the neck, chest, etc., are in many cases quite independent of any true cerebral change, but merely result from interference with sympathetic nerve influence to the intra-cranial capillaries.

Appreciating the value of the ophthalmoscope in exposing to view the beautiful and minute vessels of the choroid and retina, and bearing in mind the probability of the condition of the ophthalmic vessels being, as it were, a *reflex* of those within the cranium, Dr. Ogle thinks it is possible to seek its aid in discovering unhealthy conditions of the deep vessels of the eye in diseases of the nervous system. He furnishes the details of four interesting cases, whose history may be briefly summed up as follows:—

Case I. A man aged fifty-five; pupil of the *left* eye larger than that of the right; power of squeezing with the *left* hand much diminished; sensation of *left* arm and wrist less acute than on the opposite side; movement of *left* leg somewhat impaired; an old scar existed, along with a depression in the surface of the frontal bone over the left eye, due to a tumor, probably sebaceous, that had spontaneously broken. Had had a stroke while walking, and for a short time lost consciousness and sight, and for two or three days had no power whatever of moving the *left* arm and leg. *Examination by the ophthalmoscope*

revealed in the *left* eye, at the lower and outer part of the crimson field illuminated by the reflector, a large dark mass of substance, doubtless extravasated blood.

CASE II. Trembling of the facial muscles of the *left* side; sight dim or double, with strabismus, and distinct arcus senilis of both eyes. Had previously suffered with jerking of the muscles of the *left* side, and twitching of the *left* shoulder. *Examination by the ophthalmoscope* exhibited the presence of a slight mass of black deposit, apparently upon the retina of the *left* eye. After atropia had been twice applied to dilate the pupil, objects seemed to the left eye very much diminished, "his own hand appearing to be only of the size of a doll's hand."

CASE III. Partial ptosis of the *right* upper eyelid, with divergent strabismus of the right eyeball, and very dilated and fixed condition of the pupil. Pain, giddiness, and double vision; objects seen with the *right* eye *much larger* than they really were. *Examination by the ophthalmoscope.* Right retina much paler than the left, and hyperæsthesia of the retina of the right side—not at all so of the left—when the light of the reflecting mirror was turned upon it. Dr. Ogle has found in a case described in connection with Case 3, that a dilated and perfectly fixed pupil can be made to act almost as completely as a healthy one by long-continued use of the light thrown upon the retina by the ophthalmoscope, and advances the hypothesis that by quickening or keeping up the reflex function involved in the action of the iris under the stimulus of light, it may answer the same purpose which galvanism does when used to maintain or resuscitate the function of muscles and motor nerves in paralyzed limbs. The theory is a plausible one.

CASE IV. A well-marked illustration of intra-thoracic aneurism, situated toward the *right* side of the chest. Pupil of the *right* eye larger than on the opposite side. On the supposition that the difference depended upon some interference with the sympathetic nerve, the ophthalmoscope was employed, and numbers of darkish spots and specks (apparently the *débris* of ecchymosed blood) were visible on the field of the retina.

Dr. Ogle concludes his interesting paper by suggesting that different conditions of the cerebral capillary system might be artificially produced in lower animals for the sake of contrasting different conditions of the vessels of the choroid and retina.

### VII.—*Intermittent Phenomena, resembling Paralysis, limited to one Hand and Arm.* (American Medical Times, July 21, 1860.)

A case was admitted into the Northern Dispensary, New York, in which, at first, pain in the right hand and arm was the only symptom, soon accompanied also by a creeping and prickling sensation, as if the limb were asleep. But it was soon noticed that regular periodical phenomena supervened. For three weeks, the attacks recurred with the utmost regularity at twelve o'clock at night and four o'clock in the morning. Numbness, heat, pain, and anæsthesia of the arm came on at the first-mentioned hour, yielded to friction and warmth, and all the symptoms returned at four o'clock. She had been treated for paralysis, no case of intermittent fever having been known during the year in her neighborhood. The case was perfectly relieved by the administration of quinine.

VIII.—*Habits of Intoxication as causing a Type of Disease.* (Journal of Psychological Medicine, April, 1860.)

Intemperance and its consequences, the fruitful theme of moralists, is here discussed psychologically in its claims upon the attention of the practical physician. The writer complains that, while the immediate effects of alcoholic intoxication have been fully dwelt upon in all their mental, moral, and physical bearings upon the health and happiness of mankind, the remote morbific results of intemperance have not been so fully appreciated as to receive a legitimate position in systematic medicine. The forms of *alcoholism* which have been suggested by Dr. Léon Thomeuf in the Annales Médico-Psychologiques, 1859, p. 565, constitute perhaps as convenient a division of the subject as is practicable. He arranged them into acute, subacute, and chronic alcoholic intoxication; the first having a tendency to rapid evanescence, while the last gives rise to symptoms of insanity by determining certain structural or functional changes in the central nervous system.

The writer refers to the influence of alcoholism as a cause of insanity, as exhibited in the statistics of hospitals for the insane. Thus, of three hundred and fifty cases admitted into Charenton, in 1857 and 1858, one hundred and two were caused principally, if not solely, by the use of alcoholic drinks. After detailing the views of various authors on the nature, symptoms, and results of chronic alcoholism, and its deleterious agency in perverting the intellectual and moral faculties, the following curious and interesting, though scarcely infallible, deductions are made in regard to the influence of beer and spirits upon the mortality of the intemperate in Great Britain. The average duration of life, after the commencement of intemperate habits, is, according to one authority:*

| | |
|---|---|
| Among beer drinkers . . . . . . . | 21·7 years. |
| " spirit drinkers . . . . . . . | 16·7 " |
| " those who drink spirits and beer indiscriminately | 16·1 " |

Consequently the rate of mortality will be—

| | |
|---|---|
| Among beer drinkers . . . . . . | 4·59 per cent. yearly. |
| " spirit drinkers . . . . . . | 5·99 " " " |
| " mixed drinkers . . . . . . | 6·19 " " " |

In regard to the influence of *profession* upon alcoholism, several interesting conclusions respecting the duration of life after the commencement of intemperate habits are given. The average period, according to the same authority, is—

| | |
|---|---|
| Among mechanics, working, and laboring men . . | 18 years. |
| " traders, dealers, and merchants . . . . | 17 " |
| " professional men and gentlemen . . . . | 15 " |
| " females . . . . . . . . . . . | 14 " |

* Neison on the "Rate of Mortality among Persons of Intemperate Habits."

After discussing the treatment of chronic alcoholism, and especially the employment of the oxide of zinc as a valuable tonic, by Dr. Marcet in the Westminster Hospital, the writer suggests that "until the mental and physical phenomena of poisoning by alcohol receive an extension in the systematic teaching of medicine, and a position in our nosological arrangement in some degree pertinent to the amount of physical and moral mischief attributed to the potent liquid in ordinary life, we, the medical profession, shall probably not succeed in avoiding a habit of using phrases in reference to intoxication which to ourselves signify one thing, to the by-standers another; we shall not, therefore, succeed in convincing the public that intoxication or drunkenness the *mania*, and intoxication or drunkenness the *bad habit*, are two entirely different things—the one readily distinguishable, with care, from the other, and the one requiring the police magistrate, the other the doctor."

IX.—*On the Progressive Paralysis of the Insane.* By WILLIAM WOOD, M.D. (British and Foreign Medico-Chirurgical Review, July, 1860, p. 175.)

Dr. Wood refers first to the confusion caused by the employment of the term "paralysis," as applied to a condition in which many, "naturally expecting well-marked paralysis of sensation and motion, have overlooked the uncertain gait and tremulous articulation which to the experienced eye are as unmistakable as they are fatal symptoms of this disease." Written descriptions of the affection have often been imprecise and incomplete. It may be remarked that the term *general* paralysis is apt to mislead, from the idea that the whole body is simultaneously affected, but the truth is that this general involvement of the whole system does not really occur until the disease is near its termination, the different limbs and organs being progressively affected, and not at first simultaneously. It is from this progressiveness, if we may use the term, which is so markedly characteristic of the development and extension of the affection, that Dr. Wood proposes a change of nomenclature from General Paralysis to *Progressive Paralysis of the Insane.*

The various reasons for considering it properly a form of paralysis are detailed, as well as the main characteristics which distinguish it from ordinary examples of that affection. The condition which gives rise to the symptoms of progressive paralysis appear to be capable of arrest, although when the disease has gone on to its full establishment, there is little or no hope of averting a fatal issue. The ordinary medical attendant may often fail, when first called to a case of this nature, from his want of experience in this particular class of cases, to recognize the incipient stage of a malady which perhaps he has never seen; so that when the opinion of a special physician is taken, the disease has too often made fatal progress, and there is little or no hope of rendering effective aid. In looking back then at the history of the case, slight aberrations may be recalled, which, unless inquired into, would probably not have been particularly noticed, such as irritability of temper, irresolution of purpose, failure of memory, confusion of ideas, etc.

From facility of diversion to or from any particular cause which may be plausibly suggested, whether for good or evil, the progress toward delusions and perversions of judgment is easy and rapid, though occasionally sufficient intelligence may remain to raise a doubt in the individual's own mind, whether, after all, he may not be laboring under a delusion. But this does not continue long, and he soon finds himself unable to pursue to any length an argument or occupation of any kind. Failure of memory is one of the earliest symptoms of progressive paralysis, and when associated with alteration of manner and conduct, should be watched closely. One great difficulty in addition to the opposition of friends to the idea that insanity can possibly exist, is, according to Dr. Wood, the repugnance of the patient himself to treatment, which would seem to imply the existence of mental delusion, and so earnestly and plausibly will he oppose it, that it is difficult for the physician to persuade the family or friends that such a state of perversion has a real existence. Yet it is often this interference of friends that prevents him from being placed under circumstances which would be favorable to the arrest of the disease by affording him the benefits of professional assistance and advice.

Some of the symptoms of progressive paralysis, Dr. Wood thinks, are confounded with the incoherent condition which is known as simple mania, or they may exist without any maniacal symptoms, as a monomania, so to speak, with a generally tranquil and contented condition of mind. The course of general or progressive paralysis is not uniform, and therefore parts are not involved in the same order. The most reliable diagnostic symptom is imperfection of articulation, which is dependent upon various alterations of function in the lips, tongue, etc. The defect in ordinary stammering is dependent in most cases on the want of proper control and direction of the action of the muscles concerned in the production of speech. Proper attention may overcome this difficulty in stammering, but not so if connected with progressive paralysis, for in such cases there is muscular paralysis of the parts concerned in articulation.

Other characteristic symptoms are described, such as compression of the upper lip, quivering of the lower lip, difficulty in adjusting the oral opening, loss of muscular power in various parts of the body, increased inability to regulate the movements of the limbs, occasional convulsive attacks which differ in many respects from epileptic seizures, the attachment of undue importance and value to worthless objects, occasionally, perhaps, maniacal excitement, total abstinence from nourishment, etc. The mode of overcoming this latter difficulty, and the general treatment of those affected with progressive paralysis, are dwelt upon by Dr. Wood, but we have merely wished to give a synopsis of his views and observations on the symptomatology of the affection. It is a disease of much more frequent occurrence in the male sex than the female, may be confounded often with dementia, and is not characterized by any pathological changes which are not also found in connection with totally different symptoms. The support of the vital powers by stimulants seems to be the main indication, and morphia as well as various modes of counter-irritation may also be occasionally necessary.

Dr. Wood concludes his interesting and valuable paper by remarking: "It is unsatisfactory to reflect how little I am able to add to what is already known of this terrible malady, under which human nature appears in its most humiliating aspects, but I shall hope to have put in a somewhat clearer light than it has yet appeared to some of my readers the characteristic features of the disease, and trust that advancing science will enable us to take a more correct view of its nature, and to deal with it more successfully than we have hitherto done."

